# Assessment of Maternal Vascular Remodeling During Pregnancy in the Mouse Uterus

**DOI:** 10.3791/53534

**Published:** 2015-12-05

**Authors:** Jens Kieckbusch, Louise M. Gaynor, Francesco Colucci

**Affiliations:** ^1^Department of Obstetrics and Gynaecology, School of Clinical Medicine, University of Cambridge; ^2^Centre for Trophoblast Research, University of Cambridge

**Keywords:** Developmental Biology, Issue 106, Reproduction, Vasculature, Immunology, NK cells, Mouse, Stereology, Immunohistochemistry, Pregnancy

## Abstract

The placenta mediates the exchange of factors such as gases and nutrients between mother and fetus and has specific demands for supply of blood from the maternal circulation. The maternal uterine vasculature needs to adapt to this temporary demand and the success of this arterial remodeling process has implications for fetal growth. Cells of the maternal immune system, especially natural killer (NK) cells, play a critical role in this process. Here we describe a method to assess the degree of remodeling of maternal spiral arteries during mouse pregnancy. Hematoxylin and eosin-stained tissue sections are scanned and the size of the vessels analysed. As a complementary validation method, we also present a qualitative assessment for the success of the remodeling process by immunohistochemical detection of smooth muscle actin (SMA), which normally disappears from within the arterial vascular media at mid-gestation. Together, these methods enable determination of an important parameter of the pregnancy phenotype. These results can be combined with other endpoints of mouse pregnancy to provide insight into the mechanisms underlying pregnancy-related complications.

**Figure:**
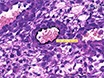


## Introduction

The exchange of nutrients, gases and waste products during eutherian gestation is mediated by the placenta. In the female reproductive tract, the uterine arteries are the main conduits of blood to the uterus. After implantation, these branch into specialized vessels called spiral arteries that coil through the decidua basalis towards the fetoplacental unit. Diameter and elasticity of these spiral arteries, which are surrounded by leukocytes, in particular uterine natural killer (uNK) cells, dictate volume and velocity of blood available to the placenta^1,2^. Correct hemodynamic changes as a result of remodeling of the spiral arteries are critical for pregnancy success.

While the underlying mechanisms differ in detail between human and murine pregnancy, the final result of the remodeling process in both species is dilated, high conductance vasculature that loses its smooth muscle layer. In mice, uNK cell-derived interferon (IFN-)γ is necessary to induce these changes at around mid-gestation^3-5^. Mice that lack either NK cells or components of the IFN-γ signaling pathway fail to undergo these changes and this is associated with reduced fetal growth^6,7^, stressing the importance of an adequate blood supply to the fetoplacental unit. During early human pregnancy, the interstitial and endovascular invasion of extravillous trophoblast and their interaction with uNK cells are important for inducing vascular changes and promoting fetal growth^8-10^. uNK cells can orchestrate human trophoblast invasion through chemokines^11^ and cytokines such as granulocyte macrophage - colony stimulating factor (GM-CSF)^12^. Shallow trophoblast invasion is associated with reduced arterial remodeling and pregnancy complications such as pre-eclampsia^9^.

This protocol is based on the pioneering work of Anne Croy who described the important role of the immune system and particularly NK-derived IFN-γ for successful vascular remodeling through immunohistochemistry and elegant transfer experiments^5,13^. Here we describe a fast and economical way to assess the remodeling status of spiral arteries. Although our lab routinely assesses this at mid-gestation (gestation day (gd)9.5)^14^, the remodeling process takes place between gd8.5 and 12.5^15^. The advent of automated slide scanners has greatly facilitated the assessment of vessel and lumen areas from sections and we found this to be a more reproducible approach than measuring vessel diameters. The addition of immunohistochemical detection of SMA allows for a straightforward validation of the results obtained from the stereological assessment. As with all stereological techniques, it is recommended to perform the assessment as a blinded experiment by at least two examiners. To this end, a randomization step can be introduced when serially cutting the samples so that the investigators assessing the slides do not know from which experimental group the samples come.

The overall goal of this protocol is to provide a reliable tool to carefully assess the remodeling of spiral arteries in mice with defined maternal and paternal genotypes, in the context of mouse models for pregnancy-related complications. The results stress the dependence on uNK cells of this process, which is essential for normal fetal growth.

## Protocol

All procedures discussed in this manuscript are in accordance with UK Home Office regulations and are approved by the Cambridge Ethical Review Panel.

### 1. Specimen Collection

1. Set up timed matings using 8-12 week old female C57BL/6 mice with adult males (up to 2 females per male) in the afternoon. Check for the presence of vaginal plugs as a sign of copulation early in the morning on the following days. The presence of a plug in the morning marks gd0.5.
2. On gd9.5, euthanize the animal by cervical dislocation and open the abdominal cavity. Note that euthanasia by CO_2_ may cause vasodilatation.
3. Use dental floss to gently ligate the uterine arteries (**Figure 1**, dashed arrows). Tie the floss around the arteries at the tip of each uterine horn, proximal to the ovaries, as well as around the cervix. Note: Do not rupture the vessels as this may result in collapsed arteries.
4. Dissect the uterus quickly using dissection scissors, excising the whole uterus distally to the three ligation points at the tip of the uterine horns and the cervix.
5. Place the uterus on a prepared polystyrene piece so that both horns are aligned along the same long axis in opposite directions from the cervix. Gently stretch it and pin into place using 2-3 25 G needles.
6. Immerse the polystyrene into a prepared 50 ml conical tube. Top up to 50 ml with 10% formalin. Fix for 5-6 hr at room temperature.
7. Discard the formalin solution and replace with 50 ml 1x phosphate buffered saline (PBS) for 5 min.
8. Excise the two uterine horns proximally to the ligation point at each end and proximally to the cervix in the center, trim away any excess fat around the implantation sites and wash twice in PBS for 5 min to remove traces of formalin.
9. Store the samples in 70% ethanol at 4 °C (for up to two weeks) until processing. CAUTION: Ethanol is highly flammable.
10. Use an automated processing system using the parameters indicated in **Table 1**. Embed the horns individually in paraffin so that they can be cut along the plane perpendicular to the long axis of the uterus. This ensures that the maximum area of each implantation site will be exposed for analysis in the middle of the block (**Figure 2A**). OPTIONAL: This is a convenient time point for randomization of the blocks if desired.
11. Using a microtome, cut serial sections of 7 µm thickness from the blocks. Stain one section at every 49 µm interval with hematoxylin and eosin (H&E, see section 2.1). Store the remaining sections at room temperature for SMA staining and other downstream applications.

**Table:** 

**Solution**	**Time**	**Temperature**
70% Ethanol	1 hr	40 °C
100% Ethanol	1 hr	40 °C
100% Ethanol	1 hr	40 °C
100% Ethanol	1 hr	40 °C
100% Ethanol	1 hr	40 °C
100% Ethanol	1 hr	40 °C
100% Ethanol	1 hr	40 °C
Xylene	1 hr	40 °C
Xylene	1 hr	40 °C
Xylene	1 hr	40 °C
Paraffin	1 hr	63 °C
Paraffin	1 hr	63 °C
Paraffin	1 hr	63 °C
Paraffin	1 hr	63 °C

**Table 1: Settings for automated tissue processing.** Overview of the setting used for the processing of uterine tissue

### 2. Stereological Assessment

1. H&E Staining
  1. Deparaffinize sections by immersing slides mounted in a slide rack into a 100% xylene bath for 10 min at room temperature. CAUTION: Xylene is flammable and a suspected carcinogen that is toxic through contact and inhalation. This work must be conducted in a fume hood, using personal protective equipment (PPE).
  2. Immerse slides in bath containing clean xylene for a further 10 min.
  3. Sequentially immerse slides in baths containing 100% ethanol, 95% ethanol, 70% ethanol and purified water for 5 min each.
  4. Immerse slides in 50% Gill No 3 hematoxylin solution, diluted with purified water, for 5 min. CAUTION: Hematoxylin is harmful by ingestion and contact to eyes. Wear appropriate PPE when handling.
  5. Wash slides in a staining trough under running tap water for 10 min.
  6. Immerse slides in 1% acid/alcohol solution (1% 10 M hydrochloric acid in 70% ethanol) for 10 sec and wash in a staining trough under running tap water.
  7. Immerse slides in eosin solution for 30 sec and wash in a staining trough under running tap water for 10 min.
  8. Within a fume hood, sequentially immerse slides in baths containing 70% ethanol, 95% ethanol and 100% ethanol for 5 min each.
  9. Immerse slides in bath containing xylene for 10 min.
  10. Mount coverslips using a xylene-based mounting medium. Dry horizontally and thoroughly in a fume hood prior to visualizing the slides under a microscope.
2. Stereological Assessment of Vessel Size and Remodeling Status
  1. For each implantation site, determine which sections are close to the midsagittal point. The chorioallantoic attachment between the fetus and developing placenta serves as a good indicator of the midsagittal point.
  2. Select 3 sections at 49 µm intervals close to the midsagittal point for the analysis. Scan selected slides. In order to avoid including veins, which have been shown to be more peripherally located in the implantation sites^16^, restrict the analysis on the central 2/4 of each implantation site (**Figure 2A**).
  3. To assess lumen size, draw around the inside of the vessels and record the area of this shape. For the total vessel size, draw around the outer wall of the vessel, including endothelial cells, pericytes and intramural leukocytes. Note: The ratio of total vessel size to lumen is a proxy for the remodeling status of a vessel. As the vessels coil through the plane that the section was cut along, there may be multiple cross-sections of the same artery in one slide. Avoid measuring the same artery multiple times.
  4. Record the measurements from the 5 vessels in each implantation site with the largest and roundest lumens. Measure each implantation site in triplicate (three sections spaced 49 µm apart from each other). The mean of these 15 measurements represents the mean diameter of the largest vessels.
  5. To avoid overrepresentation of larger vessels in some samples, measure the same number of vessels in each implantation site.

### 3. Qualitative Assessment Using Immunohistochemistry

1. Deparaffinization and Rehydration
  1. Deparaffinize sections by immersing slides mounted in a heatproof slide rack into a 100% xylene bath for 10 min at room temperature.
  2. Immerse slides in bath containing clean xylene for a further 10 min.
  3. Sequentially immerse slides in baths containing 100% ethanol, 95% ethanol and 70% and purified water for 5 min each.
2. Antigen Retrieval
  1. Prepare a 10 mM sodium citrate buffer in purified water and adjust to pH 6 using 10 M hydrochloric acid.
  2. Fill a heatproof container that can hold 600 ml with 400 ml sodium citrate buffer and place in a domestic pressure cooker containing sufficient water to submerge half of the container. Loosely attach the lid of the pressure cooker and heat until boiling.
  3. Place slide rack into the sodium citrate buffer and tightly fix the lid of the pressure cooker. Once pressurized, allow to boil for 3 min.
  4. Depressurize the pressure cooker and remove the inner container. Allow slides to cool in the sodium citrate buffer for 10 min at room temperature.
  5. Wash slides in a bath containing purified water for 5 min.
  6. Encircle the sections using a hydrophobic barrier pen.
  7. Wash slides by submerging in PBS for 5 min.
3. Blocking Endogenous Peroxidase and Non-specific Binding
  1. Incubate sections with 3% hydrogen peroxide diluted in purified water in a humidified chamber for 30 min at room temperature. CAUTION: Hydrogen peroxide is highly irritant to eyes, skin and upon ingestion. Wear appropriate PPE.
  2. Tap off excess hydrogen peroxide solution and wash slides twice by submerging in Tris buffered saline (TBS) for 2 min each.
  3. Incubate sections with mouse immunoglobulin blocking reagent from the mouse on mouse kit prepared according to manufacturer's instructions.
  4. Wash slides twice in TBS for 2 min each.
4. Immunodetection of Smooth Muscle Actin
  1. Prepare antibody diluent solution (provided in kit) and incubate with sections for 5 min at room temperature, or according to manufacturer's instructions.
  2. Dilute 1:100 mouse anti-human smooth muscle actin (DAKO M0851) in antibody diluent solution. Dilute mouse IgG2a isotype control in antibody diluent solution, ensuring that the final immunoglobulin concentration is equivalent in both solutions.
  3. Tap off excess antibody diluent solution and cover sections with either diluted antibody or isotype control solutions. Incubate in a humidified chamber for 30 min at room temperature.
  4. Wash slides twice in TBS for 2 min each.
  5. Dilute biotinylated anti-mouse secondary antibody and incubate sections for 10 min at room temperature, or according to manufacturers instructions.
  6. Wash slides once each with TBS and PBS for 2 min, respectively.
  7. Prepare 3,3'-Diaminobenzidine (DAB) solution according to manufacturer's instructions. Cover sections with DAB solution and incubate for up to 4 min, ensuring to monitor sections to avoid excess color development. CAUTION: DAB is flammable and a suspected carcinogen that is toxic through contact and inhalation. Wear appropriate PPE when handling.
  8. Immerse slides in purified water once desired intensity of staining is reached.
  9. Counterstain with 50% Gill No. 3 hematoxylin for 20 sec and wash in a staining trough under running tap water until water runs clear.
5. Dehydration and Mounting
  1. Within a fume hood, sequentially immerse slides in baths containing 70% ethanol, 95% ethanol and 100% ethanol for 5 min each.
  2. Immerse slides in bath containing xylene for 10 min.
  3. Mount coverslips using a xylene-based mounting medium. Dry horizontally and thoroughly in a fume hood prior to visualizing the slides under a microscope

## Representative Results

*Rag2^-/-^IL2rg^-/-^* mice lack all mature lymphocytes, including NK cells^17^, and their uterine arteries fail to undergo the vascular changes seen in congenic wildtype C57BL/6 (WT) mice around mid-gestation^5,14^. As a result of this reduced remodeling *Rag2^-/-^IL2rg^-/- ^*mice show significantly smaller luminal surface areas, indicative of overall smaller vessels which can be visualized on H&E-stained sections and quantified stereologically (**Figure 2**). Furthermore, the relative wall thickness (vessel to lumen ratio) is higher in the spiral arteries in these mice, suggesting reduced supply of blood and higher blood velocity.

This quantitative assessment was confirmed using immunohistochemical detection of SMA. It is characteristic for WT mice to lose most of the SMA within the vascular media of the spiral arteries by mid-gestation. If this NK cell-driven process does not take place, SMA remains in the media of the vessels and can be readily detected immunohistochemically as rings around the blood vessels (**Figure 3**).


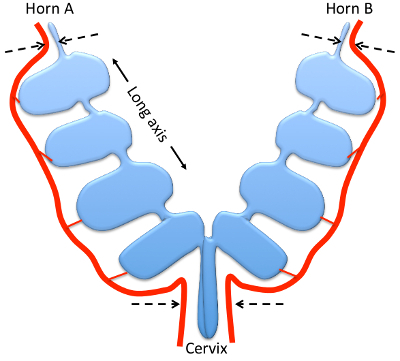
**Figure 1: Mouse uterus at gd9.5, schematic overview.** Drawing showing the relative positions of the uterine horns, cervix (blue) and main uterine arteries (red). Indicated are the long axis of the uterine horns (arrows) and the ligation points (dashed arrows). The sections for immunohistochemistry are cut along the plane of view.


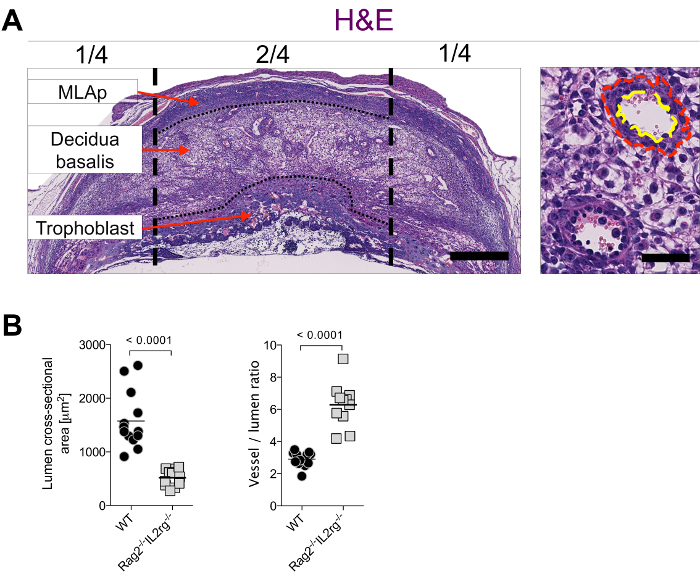


**Figure 2: Stereological assessment of arterial remodeling at mid-gestation. **(**A**) Example assessment of luminal and total vessel areas on H&E stained sections from WT females in the central 2/4 of the decidua basalis (demarcated by the vertical black dashed lines) at gd9.5. In the vessels at higher magnification, the red dashed line demarcates total vessel area, yellow dashed line demarcates lumen area. Bar = 500 µm (left), 50 µm (right). The long axis of the uterus is a horizontal line in this orientation. (**B**) Quantification of luminal areas (left panel) and ratio of luminal area to total vessel area (right panel) from WT (C57BL/6) and *Rag2^-/-^Il2rg^-/-^*mice (on C57BL/6 background). Each data point represents the mean of 15 measurements (5 measurements per section, 3 sections per implantation site). Bars represent mean of n = 11-13 implantation sites from 4 pregnancies per group; p-value from a two-tailed, unpaired Mann-Whitney test. WT: wildtype.


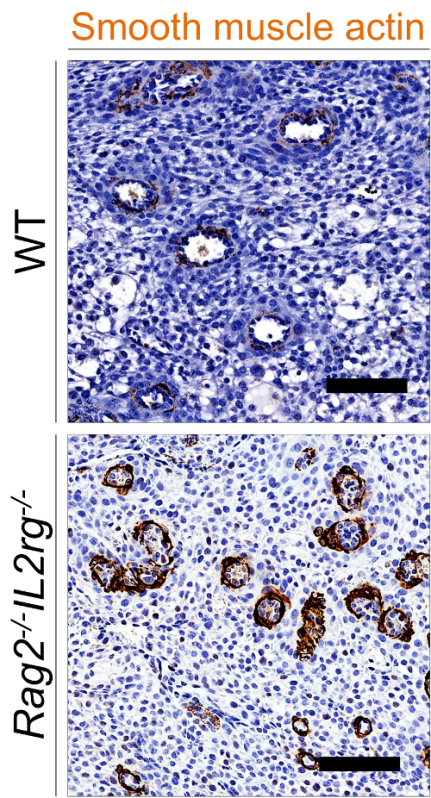
**Figure 3: Immunohistochemical detection of smooth muscle actin in the decidua basalis.** Representative SMA staining on WT (top) and *Rag2^-/-^IL2rg^-/-^* mice (on C57BL/6 background, bottom) at gd9.5. Bar = 100 µm. WT: wildtype.

## Discussion

The protocol presented here is designed to provide a reproducible method by which to assess the vascular changes that are necessary for successful pregnancy. Critical for the success of this evaluation is the quality of the tissue sections. Both accurately determining the gestational age of the implantation sites as well as reliably ligating the uterine arteries are crucial steps. Changes in parameters such as time of fixation, tissue-processing protocol, etc. may also affect the result. For an unbiased stereological assessment, it is important to measure the same number of vessels in each implantation site. It is recommended to measure 5 vessels per implantation site and each implantation site in triplicate. The mean of these 15 measurements represents the mean size of the 5 largest vessels.

Further to the data from alymphoid *Rag2^-/-^IL2rg^-/-^* mice shown here, we found this protocol useful for a number of models of inadequate maternal immune function, including one of inhibited NK cells^14^. Defects in arterial remodeling have also been shown in models of impaired trophoblast invasion^18^ and the techniques described here may be helpful to assess the vasculature in these cases. We optimized and validated this protocol for the investigation of decidual vascular remodeling at mid-gestation in mouse, but it is also conceivable to adapt this protocol to work in other model systems (*e.g.* rats) or even in other organs. While we find that the NK-dependent remodeling can be robustly assessed at gd9.5, it may be amended for other time points such as gd12.5, when the maternal vasculature is completely remodeled. At this time point, endovascular trophoblast invasion is deeper^16^ and this time point may be more suitable for investigations of the role of trophoblast for vascular changes. If additional quantitative readouts are desired, investigators can also increase the number of sections stained for SMA and assess the percentage of partially remodeled vessels within each implantation site.

A limitation of this protocol is the descriptive nature of histological examinations. Functional assays, however, are only slowly becoming available (such as Doppler ultrasound^19^). These techniques require technical expertise and much higher expenses for acquiring and maintaining equipment, but have the advantage of providing a longitudinal *in vivo* readout for the effect of the changes that can be observed histologically. A possible drawback of all stereological examinations is the subjectivity of the investigators. To this end, using two independent examiners that analyze all samples independently can help to avoid this issue. While the protocol outlined here gives insight into how much blood can reach the fetomaternal interface, it may be advantageous to combine it with the complementary approach of assessing the total amount of blood at any given time in the vasculature through plastic casts^16^.

The strength of this protocol is that it combines two independent approaches. Vascular change is first quantitatively assessed by stereology and the result is then validated qualitatively using immunohistochemistry. The reliability of the results can be further strengthened by randomizing the samples. The samples prepared for this protocol can easily be used to also investigate other parameters of pregnancy such as decidualisation, development of the mesometrial lymphoid aggregate of pregnancy (MLAp) or immunodetection of cells of interest, to rigorously assess a pregnancy phenotype at midgestation.

Arterial remodeling is a critical step for uncomplicated pregnancies in women and failure of these changes underpins the great obstetrical syndromes (GOS), namely pre-eclampsia, fetal growth restriction and stillbirth. We present here techniques to carefully assess a pregnancy phenotype in mouse models that mimic some characteristics of the pathophysiology of GOS.

## Disclosures

The authors have nothing to disclose.
